# Cost and quality-of-life impacts of community treatment orders (CTOs) for patients with psychosis: economic evaluation of the OCTET trial

**DOI:** 10.1007/s00127-020-01919-4

**Published:** 2020-07-27

**Authors:** Judit Simon, Susanne Mayer, Agata Łaszewska, Jorun Rugkåsa, Ksenija Yeeles, Tom Burns, Alastair Gray

**Affiliations:** 1 Department of Health Economics, Center for Public Health, Medical University of Vienna, Kinderspitalgasse 15/1, 1090 Vienna, Austria; 2 Department of Psychiatry, Warneford Hospital, University of Oxford and Oxford Health NHS Foundation Trust, Oxford, OX3 7JX UK; 3 Health Economics Research Centre, Nuffield Department of Population Health, University of Oxford, Richard Doll Building, Old Road Campus, Oxford, OX3 7LF UK; 4 Health Services Research Unit, Akershus University Hospital, 1478 Lørenskog, Norway; 5 Centre for Care Research, University of South-Eastern Norway, 3900 Porsgrunn, Norway

**Keywords:** Schizophrenia, Psychosis, Community treatment orders (CTOs), Economic evaluation, Cost-effectiveness, Cost-utility, Capability, OxCAP-MH, Informal care, Societal perspective

## Abstract

**Purpose:**

Current RCT and meta-analyses have not found any effect of community treatment orders (CTOs) on hospital or social outcomes. Assumed positive impacts of CTOs on quality-of-life outcomes and reduced hospital costs are potentially in conflict with patient autonomy. Therefore, an analysis of the cost and quality-of-life consequences of CTOs was conducted within the OCTET trial.

**Methods:**

The economic evaluation was carried out comparing patients (*n* = 328) with psychosis discharged from involuntary hospitalisation either to treatment under a CTO (CTO group) or voluntary status via Section 17 leave (non-CTO group) from the health and social care and broader societal perspectives (including cost implication of informal family care and legal procedures). Differences in costs and outcomes defined as quality-adjusted life years (QALYs) based on the EQ-5D-3L or capability-weighted life years (CWLYs) based on the OxCAP-MH were assessed over 12 months (£, 2012/13 tariffs).

**Results:**

Mean total costs from the health and social care perspective [CTO: £35,595 (SD: £44,886); non-CTO: £36,003 (SD: £41,406)] were not statistically significantly different in any of the analyses or cost categories. Mental health hospitalisation costs contributed to more than 85% of annual health and social care costs. Informal care costs were significantly higher in the CTO group, in which there were also significantly more manager hearings and tribunals. No difference in health-related quality of life or capability wellbeing was found between the groups.

**Conclusion:**

CTOs are unlikely to be cost-effective. No evidence supports the hypothesis that CTOs decrease hospitalisation costs or improve quality of life. Future decisions should consider impacts outside the healthcare sector such as higher informal care costs and legal procedure burden of CTOs.

**Electronic supplementary material:**

The online version of this article (10.1007/s00127-020-01919-4) contains supplementary material, which is available to authorized users.

## Introduction

In the era of deinstitutionalization of psychiatric patients, compulsory treatment in the community has become widespread internationally. Specifically, community treatment orders (CTOs) make it a legal requirement for eligible patients with severe mental illness to adhere to their treatment regimes while they live at home. With the aim to prevent repeated relapses resulting in hospital admissions, so-called ‘revolving door’ patients are targeted. Legislation for compulsory outpatient psychiatric treatment has been introduced in more than 75 jurisdictions worldwide [1]. In England and Wales, CTOs were introduced in 2008. This implementation was planned to replace existing extended use of the leave regime under Section 17 of the Mental Health Act by requiring psychiatrists to consider a CTO for leave exceeding 7 days. The introduction of CTOs was controversial, especially since scientific evidence on their effects based on rigorous methodology was lacking [2]. Obtaining such evidence was deemed particularly important in light of the complex ethical balance of personal freedom against the need for care, and the public safety issues associated with CTOs [3].


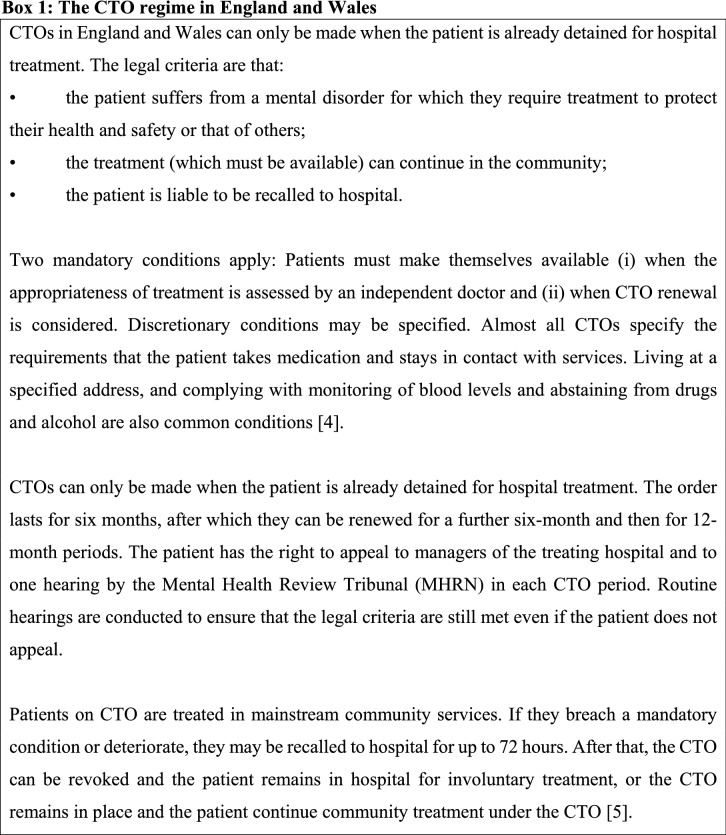


The evidence gap on effectiveness was addressed in the Oxford Community Treatment Order Evaluation Trial (OCTET) (2008–2012). The overall hypothesis was that discharge from hospital on CTO would decrease the rate of psychiatric hospital readmissions over 12 months given that patients receive similar levels of clinical contact but the length of compulsory supervision differs [4]. While OCTET found that the length of initial compulsory outpatient treatment was indeed statistically significantly different between the CTO and non-CTO groups (6 months vs 8 days), the number of readmitted patients, time to readmission and number of days hospitalised did not differ. These results are in line with two early randomised controlled trials from the US [6, 7] and with later meta-analyses of RCT evidence [8] and other types of outcome studies [9]. A 48-month follow-up study of the OCTET sample also found no difference in hospitalisation outcomes [10], and no association between CTO duration and social network size, objective social outcomes, health-related quality of life, or capabilities were found [2]. There was also no difference in community service use, with an average of two contacts per month in both arms [5].

Evidence on the overall cost-effectiveness of CTOs compared to standard care, however, is still missing in the literature [11]. While one non-randomised study from the US suggests cost reductions following CTO placement [12], no cost analysis from RCT-level studies exist. Such an assessment of the costs of related health and social care and broader resource impacts such as regular legal tribunal procedures that form part of the CTO regime, alongside outcomes such as health-related quality of life seems crucial given the originally hypothesised cost savings to the health and social care system. Given that patients on a CTO live at home, there may be changes to the costs associated with the time needed for informal family caregiving as well. At the same time, due to the increased curtailment of personal freedom, a negative impact on freedom of choice and broader wellbeing may also have been anticipated amongst patients discharged on CTO. In addition, hearings are a routine part of CTOs as legal safeguard for patients. This prospective, within-trial economic evaluation carried out alongside OCTET aimed at assessing this potential trade-off between costs and quality-of-life outcomes.

## Methods

### Study design and study population

OCTET was a multi-site, non-blinded, parallel-arm randomised controlled trial, with recruitment taking place across England between 2008 and 2011 (for further details on the clinical trial, see [4]). The clinical trial was registered with the International Standard Randomised Controlled Trial Number Register (reference: ISRCTN73110773), ethical approval was given by the Staffordshire National Health Service (NHS) Research Ethics Committee (30/10/2008, reference no. 08/H1204/131), and data were reposted in https://www.reshare.ukdataservice.ac.uk/852414/. Patient inclusion criteria were (1) aged between 18 and 65 years (i.e. the standard age range for UK adult mental health services), (2) diagnosed with psychosis, (3) currently admitted under a treatment section (Section 3 or 37) of the Mental Health Act, (4) being considered for a CTO by their clinicians (psychiatrist and Approved Mental Health Professional), (5) being able to give written and informed consent, and (6) not already participating in the study. Consenting patients were randomly assigned to be discharged from hospital on CTO (CTO group) or to voluntary status via brief Section 17 leave (non-CTO group), with the latter corresponding to standard care. In England and Wales, patients on CTO are not part of separate programmes, and patients in both arms received case-management follow-up by specialised community mental health teams.

The clinical analysis of OCTET was based on a total sample of 333 patients. Of these, five participants died during the 12-month follow-up period of reasons unrelated to treatment (CTO: *n* = 3, non-CTO: *n* = 2) and were excluded from the health economic analysis. Table 1 presents the sociodemographic and clinical characteristics of the remaining 328 patients (CTO: 163, non-CTO: 165) included in the current economic evaluation. Resource use and outcome data were collected based on face-to-face interviews with the participants at baseline, 6 months and 12 months supplemented with information from patient records.

**Table 1:** Patient characteristics at baseline

Variables	CTO (*n* = 163)		Non-CTO (*n* = 165)	
	*N*	Mean (SD) or %	*N*	Mean (SD) or %
Age (years)	163	39.89 (11.28)	165	39.30 (11.60)
Gender				
Male	109	67	112	68
Female	54	33	53	32
Missing	0	0	0	0
Marital status				
Single (never married)	122	75	122	74
Married/co-habiting	11	7	17	10
Separated/divorced	29	18	25	15
Missing	1	1	1	1
Have children				
Yes	72	44	59	36
No	90	55	105	64
Missing	1	1	1	1
Formal education (years)	161	11.73 (1.75)	163	11.98 (2.11)
Accommodation				
Independent	116	71	119	72
Supported	29	18	27	16
Homeless	17	10	18	11
Missing	1	1	1	1
Employment				
Regular paid	0	0	2	1
Voluntary/protected/sheltered	1	1	1	1
Job seekers allowance	9	6	5	3
Sickness benefit	141	87	146	88
Unemployed	8	5	5	3
Other (student/pensioner)	3	2	6	4
Missing	1	1	0	0
Religious denomination				
Christian	64	39	71	43
Jewish	3	2	2	1
Muslim	9	6	9	5
Other	27	17	15	9
None	43	26	56	34
Missing	17	10	12	7
Duration of illness (years)	158	14.57 (10.40)	162	13.98 (10.29)
Primary clinical diagnosis (ICD-10)				
Schizophrenia, schizotypal and delusional disorders	138	85	141	85
Other psychotic disorders (including bipolar)	25	15	24	15
Missing	0	0	0	0
Ethnicity				
White	100	61	102	62
Black	36	22	38	23
Asian	15	9	14	8
Mixed	12	7	11	7
Other	0	0	0	0
Missing	0	0	0	0

*N* number of participants, *SD* standard deviation, *CTO* hospital discharge on community treatment order, *non-CTO* hospital discharge on Section 17 leave

### Outcomes

The primary economic analysis was a cost-utility analysis. This was based on patient-rated measures, which were collected in face-to-face interviews with trained research assistants at baseline, 6 and 12 months. Quality-adjusted life years (QALYs) were used to measure outcomes calculated based on utilities measured by the EQ-5D-3L index on a scale where 0 represents death and 1 represents full health [13]. EQ-5D-3L is a standardised, non-disease-specific instrument designed for describing and valuing health-related quality of life (HRQoL), i.e. self-perceived health status [14]. EQ-5D-3L is based on five dimensions (mobility; self-care; usual activities; pain/discomfort; anxiety/depression) [15], and is the currently preferred measure in economic evaluations by the National Institute for Health and Care Excellence [16]. EQ-5D-3L responses were valued based on the UK tariff [17]. Changes in HRQoL over the 12 month-period were reported in QALYs gained in comparison to the baseline values [18]. Quality-of-life changes between the baseline, 6-month and 12-month measurement points were assumed to have occurred linearly. In addition, HRQoL was also assessed based on the EQ-5D Visual Analogue Scale (VAS) values as indicated on a feeling thermometer between 0 (worst imaginable health state) and 100 (best imaginable health state). In a secondary outcome analysis, capability-weighted life years (CWLY) gained were calculated using the OxCAP-MH capability wellbeing measure. The OxCAP-MH is a multi-dimensional, non-preference based, self-reported instrument based on Sen’s capability approach developed for broader quality of life measurement in mental health research [19]. Raw OxCAP-MH scores range from 16 to 80 with standardised scores ranging between 0 and 100 where 0 represents ‘no capabilities’ and 100 represents ‘full capabilities’ [2]. For the CWLYs calculation, standardised OxCAP-MH scores were transferred to a 0–1 scale.

### Resource use and costs

Resource use data collection was based on an amended version of the Client Service Receipt Inventory (CSRI) instrument [20], a widely used and validated resource use measurement instrument designed for use in mental health research. In line with the adopted analytical perspectives, collected resource use information included all hospital and community health and social services, psychiatric medication, productivity losses and the cost of informal family caregiving (see Supplementary Table 1 for detail, [21–29]). Additionally, data on the number of manager hearings and tribunals were included from the trial Case Report Forms (CRFs) as a measure of use of legal resources. All resource and cost data were extracted from patient records by trained research assistants. Due to the lack of relevant tariffs, costs associated with these legal services were not included in the cost-effectiveness analysis.

UK national-level unit costs were used to value all resource use items. To match the final year of trial follow-up, all unit costs used in the analysis refer to year 2012/13 and are expressed in British pounds (£). These together with the sources of information are reported in Supplementary Table 1. For medication costs, daily dose information was multiplied with the average (proprietary and non-proprietary) unit price for each compound reported in the British National Formulary [21]. The human capital approach was adopted to estimate lost productivity costs [30]. For study participants in employment, absent work days were multiplied by the average daily UK national salary [31, 32]. Informal care was valued based on average UK hourly salary multiplied by the number of hours family and friends spent on supporting participants as a result of their illness. Costs were assessed over 12 months, and so no discount rate was necessary.

### Analyses

In line with current NICE guidance [16], the main analysis took the perspective of the health and social care system. Additional analyses were conducted from a broader societal perspective including lost productivity and informal care.

Self-reported resource use and outcome (EQ-5D-3L) data were missing at 12 months for 42% and 58% of the participants in the CTO group and 45% and 55% of the participants in the non-CTO group, respectively (Table 2, Supplementary Table 3). Missing data were supplemented from patient records or dealt with using multiple imputation (chained predictive mean matching). In the latter case, missing values were replaced with values predicted based on randomisation group, age, gender, main clinical diagnosis, illness duration and length of inpatient stay as covariates [33]. The number of imputations sets was 30 for costs and EQ-5D and 50 for the OxCAP-MH index to match the percentage of incomplete cases [34]. Results of the health economic analysis are presented separately for the full dataset (*n* = 328) as main analysis and the complete cases datasets (health and social care perspective: *n* = 121; broader societal perspective: *n* = 102) as secondary analyses. Further sensitivity analyses were carried out to assess the impact of the original linear assumption on quality-of-life changes.

**Table 2:** Health-related quality of life and capability wellbeing outcomes at baseline, 6 months and 12 months including QALYs and CWLYs gained during 12 months in comparison to baseline

	*N*	EQ-5D-3L utility						*N*	EQ-5D VAS						*N*	OxCAP-MH						QALYs gained			CWLYs gained		
		Baseline		6 months		12 months			Baseline		6 months		12 months			Baseline		6 months		12 months		Baseline—12 months			Baseline—12 months		
		Mean	(SD)	Mean	(SD)	Mean	(SD)		Mean	(SD)	Mean	(SD)	Mean	(SD)		Mean	(SD)	Mean	(SD)	Mean	(SD)	*N*	Mean	(SD)	*N*	Mean	(SD)
Imputed full dataset																											
CTO	163	0.73	0.26	0.78	0.23	0.71	0.30	163	65.72	21.37	72.82	13.92	68.16	18.80	163	67.16	11.66	70.99	9.41	70.14	10.18	163	0.020	0.19	163	0.027	0.085
Non-CTO	165	0.70	0.27	0.72	0.22	0.74	0.25	165	64.85	22.73	69.59	16.31	71.60	18.29	165	65.06	10.17	67.36	9.06	67.82	9.95	165	0.015	0.20	165	0.018	0.081
Difference	328	0.03	0.03	0.07*	0.02	− 0.02	0.03	238	0.87	2.44	3.23	1.68	− 3.44	2.05	328	2.10	1.21	3.63*	1.02	2.32*	1.11	328	0.006	0.02	328	0.008	0.009
Complete case analysis																											
CTO	69	0.77	0.24	0.82	0.28	0.80	0.26	65	69.22	21.96	75.74	15.77	71.95	19.62	32	71.44	14.65	75.29	12.16	74.90	10.47	69	0.031	0.19	32	0.028	0.076
Non-CTO	74	0.68	0.31	0.70	0.29	0.75	0.29	71	64.83	25.22	68.94	22.71	71.52	21.95	35	66.43	13.73	67.54	15.15	70.27	11.23	74	0.029	0.23	35	0.015	0.109
Difference	143	0.09	0.05	0.12*	0.05	0.05	0.05	136	4.38	4.07	6.79*	3.38	0.43	3.58	67	5.01	3.47	7.75*	3.38	4.63*	2.66	143	0.003	0.04	67	0.013	0.023

*N* number of participants, *SD* standard deviation, *CTO* hospital discharge on community treatment order, *non-CTO* hospital discharge on Section 17 leave, *VAS* Visual Analogue Scale, *QALYs* quality-adjusted life years, *CWLYs* capability-weighted life years

*p < 0.05

Results are reported as means with standard deviations (SD) or as mean differences with 95% confidence intervals. A regression framework was used for comparing the differences in mean costs and effects with *p* < 5% considered as statistically significant. Non-parametric bootstrapping [35] of the cost and effectiveness data was applied to generate a joint distribution of the mean incremental costs and effects and to calculate the 95% confidence intervals of the incremental cost-effectiveness ratio (ICER). Uncertainty around the main cost-effectiveness estimates was represented by cost-effectiveness acceptability curves (CEACs) based on the net benefit approach [36, 37]. CEACs indicate the probability that each option is cost-effective across a range of different maximum costs per QALY gained ceiling ratios that a decision-maker might be willing to pay for an additional unit of improvement in outcomes.

All analyses were conducted according to the intention-to-treat principle and carried out in Microsoft^®^ Excel and Stata^®^ (StataCorp, College Station, TX, USA).

## Results

### Outcomes

The mean values of the EQ-5D-3L utility, EQ-5D VAS and the OxCAP-MH capability index at baseline, 6 months and 12 months are summarised in Table 2. Table 2 also reports changes in EQ-5D levels from baseline in the form of QALYs gained and changes in OxCAP-MH levels from baseline in the form of CWLYs gained during the 12-month follow-up. No significant difference could be detected between the CTO and non-CTO groups with regards to QALYs gained or CWLYs gained over the 12 months (Table 2).

### Resource use and costs

Mean observed resource use information on health and social care service utilisation is presented in Supplementary Table 2. Regarding employment, one participant in the CTO group and five participants in the non-CTO group had any period of employment/self-employment during the 12-month follow-ups with only one of these participants reporting some absence due to sick leave. There were significantly more manager hearings and tribunals in the CTO group in comparison with the non-CTO group (Table 3).

**Table 3:** Observed use of manager hearings and tribunals

	CTO			Non-CTO			CTO vs. Non-CTO		
	Mean	(SD)	*N*	Mean	(SD)	*N*	Mean difference	95% CI	
Imputed full dataset									
Number of tribunals (MHRT)	0.552	(0.72)	163	0.293	(0.54)	165	0.259*	0.12	0.40
Number of manager hearings	0.350	(0.55)	163	0.221	(0.53)	165	0.128*	0.01	0.25
Complete case analysis									
Number of tribunals (MHRT)	0.552	(0.72)	163	0.293	(0.54)	164	0.259*	0.12	0.40
Number of manager hearings	0.350	(0.55)	163	0.220	(0.53)	164	0.130*	0.01	0.25

*N* number of participants, *SD* standard deviation, *CI* confidence interval. *CTO* hospital discharge on community treatment order, *non-CTO* hospital discharge on Section 17 leave, *MHRT* Mental Health Review Tribunal. *p < 0.05

Table 4 presents the mean health and social care costs and productivity and informal care costs per participant over the 12-month follow-up based on all cases with multiple imputation for missing data. Mean per patient total health and social care costs were estimated at £35,959 (SD: £44,886) in the CTO group and £36,003 (SD: £41,406) in the non-CTO group. With more than 85% of annual health and social care costs, mental health hospitalisations were the biggest cost driver. Results of the secondary cost analyses based on complete cases are presented in Supplementary Table 3. No significant differences between the CTO and non-CTO group could be found in any of the included health and social care cost categories, either in the main analysis or in the complete case analyses.

**Table 4:** Mean health and social care costs and lost productivity and informal care costs per participant over the 12-month period based on all cases with multiple imputation for missing data (£, year 2012/13 tariffs)

	CTO			Non-CTO			CTO vs. Non-CTO		
	Mean	(SD)	*N*	Mean	(SD)	*N*	Mean difference	95% CI	
Imputed full dataset									
Total medication costs	1265.95	(1198.90)	163	1432.48	(1487.47)	165	− 166.53	− 458.74	125.68
Oral medication	920.90	(1064.80)	163	895.55	(997.22)	165	25.35	− 197.98	248.67
Depot medication	345.05	(778.43)	163	536.93	(1281.88)	165	− 191.87	− 421.08	37.34
Total other health and social care costs	34,693.07	(44,928.06)	163	34,570.71	(41,382.25)	165	122.36	− 9,228.61	9,473.33
Mental health community/outpatient	2417.11	(2019.22)	163	2,280.80	(2,424.34)	165	136.32	− 346.30	618.94
Mental health inpatient	30,655.11	(44,862.35)	163	30,393.42	(41,341.95)	165	261.69	− 9,077.69	9,601.07
Non-mental health outpatient	92.60	(281.59)	163	125.81	(238.42)	165	− 33.21	− 89.71	23.29
Non-mental health inpatient	78.64	(332.40)	163	312.38	(1,439.77)	165	− 233.74	− 459.27	− 8.21
Primary care	149.95	(221.39)	163	207.76	(377.03)	165	− 57.81	− 124.63	9.01
Social care	1299.66	(1707.99)	163	1250.54	(1,885.22)	165	49.12	− 340.10	438.34
Total health and social care costs	35,959.02	(44,886.29)	163	36,003.19	(41,406.39)	165	− 44.17	− 9392.90	9304.56
Indirect costs	6138.40	(13,752.46)	163	2992.59	(8407.44)	165	3145.81*	675.40	5616.22
Lost productivity	0.00	(0.00)	163	28.31	(363.64)	165	− 28.31	− 83.79	27.18
Informal care	6138.40	(13,752.46)	163	2964.28	(8404.91)	165	3174.12*	703.91	5644.33
Total societal costs	42,097.43	(45,977.40)	163	38,995.79	(41,475.12)	165	3101.64	− 6378.23	12,581.51

*N* number of participants, *SD* standard deviation, *CI* confidence interval, *CTO* hospital discharge on community treatment order, *non-CTO* hospital discharge on Section 17 leave

*p < 0.05

Participants in the CTO group had significantly higher mean in terms of the cost of informal family caregiving than patients in the non-CTO group (£6138 vs. £2993) (Table 4).

### Cost-effectiveness

There were no significant changes over time in any of the outcome measures, nor could a significant effect difference be detected between the CTO and the non-CTO groups in terms of QALYs gained (full imputed dataset: 0.006; 95% CI − 0.04 to 0.05; complete cases: 0.003, 95% CI − 0.07 to 0.07) or CWLYs gained (full imputed dataset: 0.008, 95% CI − 0.01 to 0.03; complete cases: 0.013, 95% CI − 0.03 to 0.06). Differences in mean total health and social care costs (full imputed dataset: £− 44, 95% CI − 9393 to 9305; complete cases: £5388, 95% CI − 7107 to 17,883) or mean total societal costs (full imputed dataset: £3102, 95% CI − 6378 to 12,582; complete cases: £7067, 95% CI − 7219 to 21,353) between the groups were also non-significant.

**Fig. 1:**
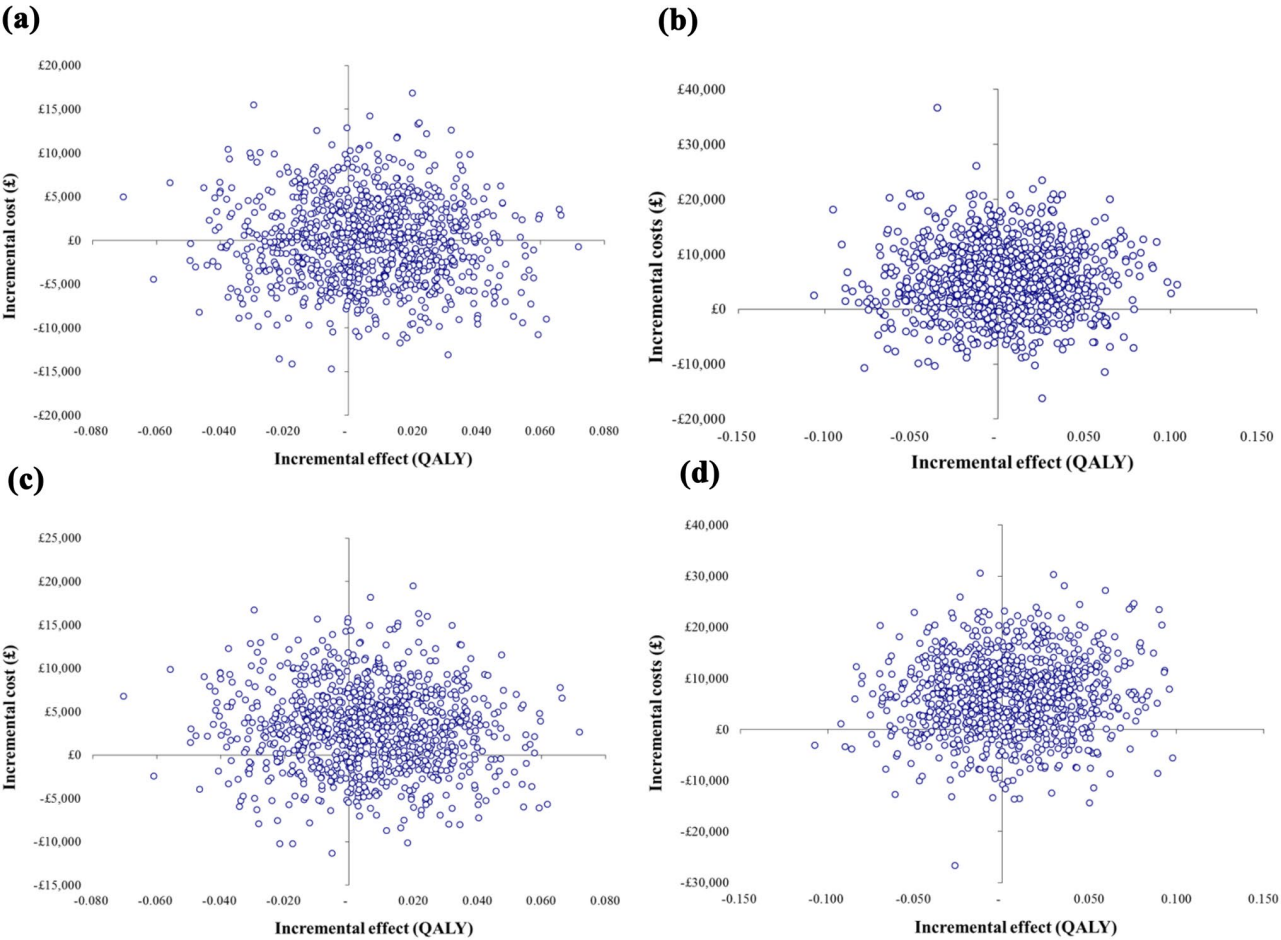
Bootstrapped mean differences in costs and effects of CTO vs*.* non-CTO: **a** health and social care costs, imputed full dataset (*n* = 328); **b** health and social care costs, complete case analysis (*n* = 121); **c** societal costs and effects, imputed full dataset (*n* = 328); **d** societal costs and effects, complete case analysis (*n* = 102)

Figure 1 shows the scatterplots of the bootstrapped incremental cost and effectiveness pairs between the CTO and non-CTO groups for the different analytical scenarios. As the points in the scatterplot fall within all four quadrants of the cost-effectiveness plane, major uncertainties remain around the cost-effectiveness of CTO vs. non-CTO, and no clear conclusion can be drawn in this respect. Supplementary Fig. 1 illustrates the uncertainty around the ICER in the form of CEACs. These clearly demonstrate that in any of the analytical scenarios the probability of CTO being cost-effective remains at around 50% or below at the currently considered maximum UK cost-effectiveness threshold value of £20,000–£30,000/QALY [16].

In the sensitivity analysis, changing the assumption that quality-of-life changes between follow-up points occurred linearly did not change any of the above results or conclusions. The QALY differences between CTO and non-CTO groups for the full imputed dataset were 0.008 (95% CI − 0.10, 0.08; *p* = 0.87) and 0.017 (95% CI − 0.03, 0.07; *p* = 0.49), when assuming changes occurred at the beginning or at the end, respectively.

## Discussion

This economic evaluation is the first prospectively designed, comprehensive analysis of the cost and HRQoL and capability wellbeing consequences of CTOs over a 12-month follow-up period in comparison to non-CTOs both from the health and social care perspective and a broader societal perspective. We found no evidence that CTOs have any significant impact on how patients rated their HRQoL or capability well-being. This finding is in line with the measures of symptoms and functioning in OCTET, i.e. no change over time in either group and no difference between groups [1]. No difference in objective levels of coercion between the CTO and non-CTO group could be identified either [1].

Much of the earlier debate about the introduction of CTOs focused on their potential impact on ‘keeping patients in contact’ with services. Earlier studies had identified high rates of drop out in psychosis patients [38, 39]. It appears that, in the UK at least, assertive follow-up of severely psychotic patients has improved significantly. In the OCTET outcome paper [1] clinical teams were found to keep good contact with both CTO and non-CTO patients (an average of more than two contacts per month over the study period). This level of regular contact and low level of loss to follow-up were both found in the 3-year follow-up [10] and may go some way to explain the absence of differences in both clinical, patient-reported and cost outcomes.

Although there are growing concerns about the responsiveness of the EQ-5D in psychosis, CTOs were anticipated to affect at least some of the health domains it measures. While the OxCAP-MH is a newer and less widely used quality-of-life measurement instrument, it has been successfully validated in several studies for different mental health disorders including psychotic patients and has been designed to measure broader well-being and freedom aspects specifically as discussed earlier [40–43]. Given the consistency of the measures in detecting no difference between the groups in terms of outcomes, it may simply be the case that no significant difference emerged in how the patients experienced their quality of life over the 12-month observation period rather than the measures having no sensitivity to change. The two other RCTs conducted to date similarly found no effect of CTOs on patient-rated quality-of-life [6, 7] and no improvements were found when following up OCTET patients over 4 years [2]. Our findings are also supported by the lack of effect on other patient-rated measures that are likely to affect quality of life such as social and clinical relationships, satisfaction with services and experienced coercion [2, 44].

Regarding costs, no difference could be detected in the health and social care costs of patients in the CTO versus non-CTO groups. Specifically, no evidence was found to support the hypothesis that CTOs result in lower hospitalisation costs. While CTOs significantly increased the cost of family caregiving, the overall difference in all costs between the two groups remained non-significant. However, if it had been possible to include the costs of the additional legal procedures in the CTO group, a significant difference could potentially have emerged further reducing the likelihood of CTOs being cost-effective measures. Moreover, a recent meta-analysis suggests that CTOs might increase the use of community services [9], which could increase their costs.

## Limitations

Although we could not reach all patients for face-to-face interviews after the baseline interview, and there were some missing information on outcomes and resource use at follow-ups, we supplemented missing resource use information from medical notes including data on legal procedures. Any remaining missingness, mainly in outcomes was handled through multiple imputation. Alternative analyses on the full imputed dataset and on the complete cases only dataset were presented, showing similar results and conclusions. Non-parametric bootstrapping was applied to determine the overall uncertainty in the cost-effectiveness results. In our costing, we were not able to value the legal consequences of the alternatives in monetary terms due to the lack of relevant up-to-date unit cost estimates and the lack of feasibility to develop these. Had we included legal costs also in our analysis from a broader perspective, we might have found evidence of significantly increased overall societal costs by CTOs, strengthening the evidence to reject the hypothesis that CTOs are cost-effective arrangements.

## Conclusions

The current economic evaluation does not support the hypothesis that CTOs reduce health and social services costs due to decreased hospital re-admissions, nor that CTOs are associated with improved HRQoL or broader well-being. Likewise, there was no difference in the cost of community service use. Although there is substantial uncertainty about the exact incremental cost-effectiveness of CTOs compared to non-CTOs, none of the sensitivity analyses changed the conclusion that CTOs are unlikely to be cost-effective. In line with the increasing consideration of so-called inter-sectoral costs and benefits of health care services, programmes and interventions [45], however, it is vital that the findings on the increased informal care, legal procedure burdens, and other indirect costs of CTOs are taken into consideration in future decision-making.

## Electronic supplementary material

Below is the link to the electronic supplementary material.

Supplementary file1 (DOCX 168 kb)
