# Supplementary material for: Cost and quality-of-life impacts of community treatment orders (CTOs) for patients with psychosis: economic evaluation of the OCTET trial
Source: Soc Psychiatry Psychiatr Epidemiol. 2020 Jul 27;56(1):85–95. doi: 10.1007/s00127-020-01919-4 (PMC7847440; doi:10.1007/s00127-020-01919-4)
Supplement: Supplementary file 1 — Supplementary file1 (DOCX 168 kb) [file 127_2020_1919_MOESM1_ESM.docx]

| **Supplementary Table 1: Unit costs (£, year 2012/13 tariffs)** | | | |
| --- | --- | --- | --- |
| **Resource use** | **Unit costs (£)** | **Unit** | **Source of estimate** |
| **Psychiatric medication** |  |  |  |
| Oral medication | various | per daily dose (mg) | BNF 64 (September 2012) [21] |
| Depot medication | various | per administered depot (mL/mg) | BNF 64 (September 2012) [21] |
| **Mental health community/outpatient** |  |  |  |
| Community mental health nurse – phone contact | 6.7 | per contact | Curtis (2012) [22] |
| Community mental health nurse - face-to-face contact in NHS setting | 16.8 | per visit | Curtis (2012) [22] |
| Community mental health nurse - face-to-face contact in community | 35.6 | per visit | Curtis (2012) [22] |
| Psychiatrist - phone contact | 24.4 | per contact | NHS reference costs 2012-13 [23], Curtis (2012) [22] |
| Psychiatrist - face-to-face contact in NHS setting | 40.0 | per visit | NHS reference costs 2012-13 [23], Curtis (2012) [22] |
| Psychiatrist - face-to-face contact in community | 102.2 | per visit | NHS reference costs 2012-13 [23], Curtis (2012) [22] |
| Psychologist - phone contact | 16.1 | per contact | Curtis (2012) [22] |
| Psychologist - face-to-face contact in NHS setting | 136.0 | per visit | Curtis (2012) [22] |
| Psychologist - face-to-face contact in community | 173.5 | per visit | Curtis (2012) [22] |
| Drug/Alcohol service worker - face-to-face contact in NHS setting | 48.0 | per visit | Curtis (2012) [22] |
| Drug/Alcohol service worker - face-to-face contact in community | 122.7 | per visit | Curtis (2012) [22] |
| Other secondary care worker (e.g. occupational therapist) - face-to-face contact in NHS setting | 51.9 | per visit | Curtis (2011) [24]^1^ |
| Other secondary care worker (e.g. occupational therapist) - face-to-face contact in community | 66.1 | per visit | Curtis (2011) [24]^1^ |
| Day centre (groups/programs, non-health care staff) | 37.0 | per session | Curtis (2012) [22] |
| Day hospital (group therapies etc., health care staff, regular) | 100.0 | per day | Curtis (2012) [22] |
| Drop-in centre (including street agencies) (informal) | 37.0 | per session | Curtis (2012) [22] |
| Self-help group/support group | 59.0 | per session | Curtis (2012) [22] |
| Attendance in other facility (social club, dinner club) | 6.9 | per visit | Community Accountancy Self Help (2008)^1^ [25] |
| **Mental health inpatient** |  |  |  |
| Psychiatric hospital inpatient general ward | 345.9 | per day | Curtis (2012) [22] |
| Psychiatric hospital inpatient PICU (Psychiatric Intensive Care Unit) | 669.3 | per day | Curtis (2012) [22] |
| **Non-mental health outpatient** |  |  |  |
| Alternative therapies (NHS) - face-to-face contact in NHS setting | 43.8 | per visit | NHS Choices [26] |
| Alternative therapies (NHS) - face-to-face contact in community | 43.8 | per visit | NHS Choices [26] |
| Alternative therapies (private) - face-to-face contact in private health care facility | 67.0 | per visit | Private Healthcare Tariff (2012) [27] |
| Alternative therapies (private) - face-to-face contacts in community | 67.0 | per visit | Private Healthcare Tariff (2012) [27] |
| Day patient hospital attendance/ Accident and emergency attendance | 117.0 | per attendance | NHS reference costs 2012-13 [23] |
| Other medical/surgical outpatient visits | 3.0-204.0 | per visit | NHS reference costs 2012-13 [23] |
| **Non-mental health inpatient** |  |  |  |
| Other medical/surgical inpatient department | 61.0-1,964.0 | per day | Scottish National Tariff 2012/2013 [28], NHS reference costs 2012-13 [23] |
| **Primary care** |  |  |  |
| GP - phone contact | 22.0 | per contact | Curtis (2012) [22] |
| GP - face-to-face contact in NHS setting | 38.0 | per visit | Curtis (2012) [22] |
| GP - face-to-face contact in community | 92.0 | per visit | Curtis (2012) [22] |
| Practice nurse - phone contact | 4.5 | per contact | Curtis (2012) [22] |
| Practice nurse - face-to-face contact in NHS setting | 11.6 | per visit | Curtis (2012) [22] |
| Practice nurse - face-to-face contact in community | 23.9 | per visit | Curtis (2012) [22] |
| Other primary care worker (e.g. dietician or nutritionist) - phone contact | 3.6 | per contact | Curtis (2012) [22] |
| Other primary care worker (e.g. dietician or nutritionist) - face-to-face contact in NHS setting | 30.0 | per visit | Curtis (2012) [22] |
| Other primary care worker (e.g. dietician or nutritionist) - face-to-face contact in community | 38.3 | per visit | Curtis (2012) [22] |
| **Social care** |  |  |  |
| Community support worker - face-to-face contact in NHS setting | 8.1 | per visit | Curtis (2012) [22] |
| Community support worker - face-to-face contact in community | 10.3 | per visit | Curtis (2012) [22] |
| Social worker - phone contact | 18.5 | per contact | Curtis (2012) [22] |
| Social worker - face-to-face contact in NHS setting | 52.0 | per visit | Curtis (2012) [22] |
| Social worker - face-to-face contact in community | 66.3 | per visit | Curtis (2012) [22] |
| Home help/home care worker - phone contact | 5.7 | per contact | Curtis (2012) [22] |
| Home help/home care worker - face-to-face contact in NHS setting | 25.5 | per visit | Curtis (2012) [22] |
| Home help/home care worker - face-to-face contact in community | 25.5 | per visit | Curtis (2012) [22] |
| Housing worker - phone contact | 2.6 | per contact | Assuming national average salary, ONS (2013) [29] |
| Housing worker - face-to-face contact in NHS setting | 5.2 | per visit | Assuming national average salary, ONS (2013) [29] |
| Housing worker - face-to-face contact in community | 6.6 | per visit | Assuming national average salary, ONS (2013) [29] |
| Voluntary/Charity worker (e.g. advocacy) - phone contact | 2.6 | per contact | Assuming national average salary, ONS (2013) [29] |
| Voluntary/Charity worker (e.g. advocacy) - face-to-face contact in NHS setting | 5.2 | per visit | Assuming national average salary, ONS (2013) [29] |
| Voluntary/Charity worker (e.g. advocacy) - face-to-face contact in community | 6.6 | per visit | Assuming national average salary, ONS (2013) [29] |
| **Indirect costs** |  |  |  |
| Lost productivity | 116.8 | per day | Assuming national average salary, ONS (2013) [29] |
| Informal care | 15.6 | per hour | Assuming national average salary, ONS (2013) [29] |

Note: ^1^Adjusted for inflation based on the hospital and community health services (HCHS) pay and prices index [22].

**Supplementary Table 2: Observed resource utilisation of health and social care services**

|  | **6 months** | | | | | | | **12 months** | | | | | |
| --- | --- | --- | --- | --- | --- | --- | --- | --- | --- | --- | --- | --- | --- |
|  | | **CTO** | | | **Non-CTO** | | | **CTO** | | | **Non-CTO** | | |
|  | | **N** | **Mean** | **(SD)** | **N** | **Mean** | **(SD)** | **N** | **Mean** | **(SD)** | **N** | **Mean** | **(SD)** |
| Community mental health nurse - phone contacts | | 121 | 0.90 | 3.36 | 109 | 0.97 | 3.14 | 120 | 1.45 | 4.14 | 110 | 1.23 | 4.02 |
| Community mental health nurse - face-to-face contacts | | 121 | 15.26 | 22.88 | 110 | 11.52 | 19.06 | 120 | 14.51 | 20.23 | 110 | 12.10 | 25.51 |
| Community mental health nurse - face-to-face contacts in NHS setting | | 121 | 4.68 | 11.78 | 110 | 3.60 | 6.32 | 119 | 3.66 | 6.96 | 110 | 3.34 | 9.63 |
| Community mental health nurse - face-to-face contacts in community | | 121 | 10.58 | 18.53 | 110 | 8.02 | 18.97 | 118 | 11.05 | 19.99 | 110 | 8.76 | 21.29 |
| Psychiatrist - phone contacts | | 119 | 0.13 | 0.72 | 110 | 0.10 | 0.66 | 116 | 0.12 | 0.75 | 109 | 0.00 | 0.05 |
| Psychiatrist - face-to-face contacts | | 119 | 6.00 | 7.62 | 110 | 4.45 | 6.06 | 116 | 4.00 | 5.69 | 109 | 3.74 | 5.34 |
| Psychiatrist - face-to-face contacts in NHS setting | | 119 | 5.32 | 7.37 | 109 | 3.99 | 5.99 | 116 | 3.60 | 5.76 | 108 | 3.31 | 5.26 |
| Psychiatrist - face-to-face contacts in community | | 119 | 0.67 | 2.71 | 109 | 0.50 | 1.96 | 116 | 0.40 | 1.35 | 108 | 0.46 | 1.65 |
| Psychologist - phone contacts | | 117 | 0.01 | 0.09 | 112 | 0.00 | 0.00 | 117 | 0.01 | 0.09 | 109 | 0.01 | 0.10 |
| Psychologist - face-to-face contact | | 117 | 0.29 | 1.17 | 112 | 1.15 | 4.47 | 117 | 1.09 | 4.00 | 109 | 0.58 | 2.42 |
| Psychologist - face-to-face contacts in NHS setting | | 117 | 0.26 | 1.09 | 112 | 1.15 | 4.47 | 117 | 1.07 | 3.98 | 109 | 0.39 | 1.53 |
| Psychologist - face-to-face contacts in community | | 117 | 0.03 | 0.28 | 112 | 0.00 | 0.00 | 117 | 0.03 | 0.21 | 109 | 0.19 | 1.92 |
| Drug/Alcohol service worker - face-to-face contacts | | 118 | 0.18 | 1.10 | 112 | 0.15 | 1.17 | 117 | 0.71 | 3.43 | 110 | 0.62 | 3.50 |
| Drug/Alcohol service worker - face-to-face contacts in NHS setting | | 118 | 0.14 | 1.08 | 112 | 0.15 | 1.17 | 117 | 0.36 | 2.47 | 110 | 0.13 | 1.02 |
| Drug/Alcohol service worker - face-to-face contacts in community | | 118 | 0.03 | 0.22 | 112 | 0.00 | 0.00 | 117 | 0.35 | 2.43 | 110 | 0.49 | 3.36 |
| Other secondary care worker (e.g. occupational therapists) - face-to-face contacts | | 118 | 2.86 | 8.10 | 111 | 1.21 | 4.45 | 118 | 1.13 | 3.93 | 109 | 1.28 | 4.40 |
| Other secondary care worker (e.g. occupational therapists) - face-to-face contacts in NHS setting | | 118 | 2.53 | 7.54 | 111 | 0.90 | 3.61 | 118 | 0.60 | 2.56 | 109 | 0.93 | 3.92 |
| Other secondary care worker (e.g. occupational therapists) - face-to-face contacts in community | | 118 | 0.33 | 3.23 | 111 | 0.31 | 2.40 | 118 | 0.50 | 2.86 | 109 | 0.36 | 1.93 |
| Community support worker - face-to-face contacts | | 118 | 1.72 | 6.78 | 111 | 4.30 | 16.24 | 118 | 3.46 | 16.91 | 109 | 4.73 | 21.70 |
| Community support worker - face-to-face contacts in NHS setting | | 118 | 0.25 | 2.40 | 111 | 0.27 | 2.48 | 118 | 0.08 | 0.67 | 109 | 0.08 | 0.59 |
| Community support worker - face-to-face contacts in community | | 118 | 1.47 | 6.40 | 111 | 4.03 | 16.11 | 118 | 3.38 | 16.91 | 109 | 4.65 | 21.70 |
| GP - phone contacts | | 120 | 0.01 | 0.10 | 112 | 0.10 | 0.55 | 117 | 0.01 | 0.10 | 108 | 0.00 | 0.05 |
| GP - face-to-face contacts | | 120 | 1.41 | 2.84 | 112 | 2.12 | 4.38 | 117 | 1.62 | 4.90 | 108 | 2.21 | 4.84 |
| GP - face-to-face contacts in NHS setting | | 120 | 1.36 | 2.82 | 112 | 1.91 | 3.86 | 117 | 1.61 | 4.90 | 108 | 1.93 | 4.64 |
| GP - face-to-face contacts in community | | 120 | 0.05 | 0.55 | 112 | 0.21 | 2.27 | 117 | 0.01 | 0.09 | 108 | 0.29 | 1.74 |
| Practice nurse - phone contacts | | 119 | 0.00 | 0.00 | 112 | 0.01 | 0.09 | 117 | 0.00 | 0.00 | 108 | 0.00 | 0.00 |
| Practice nurse - face-to-face contacts | | 119 | 0.94 | 2.70 | 112 | 1.04 | 3.34 | 117 | 0.87 | 2.47 | 108 | 0.92 | 3.31 |
| Practice nurse - face-to-face contacts in NHS setting | | 119 | 0.93 | 2.70 | 112 | 1.03 | 3.35 | 117 | 0.87 | 2.47 | 108 | 0.74 | 2.81 |
| Practice nurse - face-to-face contacts in community | | 119 | 0.01 | 0.09 | 112 | 0.01 | 0.09 | 117 | 0.00 | 0.00 | 108 | 0.18 | 1.83 |
| Other primary care worker (e.g. dietician or nutritionist) - phone contacts | | 119 | 0.00 | 0.00 | 112 | 0.00 | 0.00 | 118 | 0.00 | 0.00 | 108 | 0.00 | 0.00 |
| Other primary care worker (e.g. dietician or nutritionist) - face-to-face contacts | | 119 | 0.08 | 0.57 | 112 | 0.05 | 0.40 | 118 | 0.13 | 1.20 | 108 | 0.07 | 0.43 |
| Other primary care worker (e.g. dietician or nutritionist) - face-to-face contacts in NHS setting | | 119 | 0.07 | 0.56 | 112 | 0.05 | 0.40 | 118 | 0.12 | 1.20 | 108 | 0.07 | 0.43 |
| Other primary care worker (e.g. dietician or nutritionist) - face-to-face contacts in community | | 119 | 0.01 | 0.09 | 112 | 0.00 | 0.00 | 118 | 0.01 | 0.09 | 108 | 0.00 | 0.00 |
| Social worker - phone contacts | | 117 | 0.57 | 3.31 | 111 | 0.43 | 1.90 | 118 | 0.51 | 3.26 | 109 | 1.01 | 4.51 |
| Social worker - face-to-face contacts | | 118 | 3.73 | 7.28 | 111 | 3.77 | 7.87 | 120 | 3.58 | 7.78 | 109 | 3.90 | 7.42 |
| Social worker - face-to-face contacts in NHS setting | | 117 | 1.07 | 3.32 | 111 | 1.20 | 3.76 | 119 | 1.00 | 3.54 | 109 | 1.30 | 3.24 |
| Social worker - face-to-face contacts in community | | 117 | 2.68 | 6.28 | 111 | 2.58 | 7.07 | 119 | 2.61 | 7.29 | 109 | 2.60 | 6.51 |
| Home help/home care worker - phone contacts | | 118 | 0.41 | 4.42 | 111 | 0.00 | 0.00 | 117 | 0.41 | 4.44 | 109 | 0.00 | 0.00 |
| Home help/home care worker - face-to-face contacts | | 118 | 10.99 | 40.40 | 111 | 12.59 | 40.99 | 117 | 14.58 | 48.25 | 109 | 14.85 | 51.35 |
| Home help/home care worker - face-to-face contacts in NHS setting | | 118 | 1.29 | 14.04 | 111 | 0.00 | 0.05 | 117 | 2.84 | 21.72 | 109 | 0.33 | 3.35 |
| Home help/home care worker - face-to-face contacts in community | | 118 | 9.69 | 38.22 | 111 | 12.58 | 40.99 | 117 | 11.74 | 43.86 | 109 | 14.53 | 51.33 |
| Housing worker - phone contacts | | 119 | 0.05 | 0.55 | 111 | 0.00 | 0.00 | 117 | 0.14 | 1.17 | 109 | 0.04 | 0.30 |
| Housing worker - face-to-face contacts | | 119 | 2.65 | 15.92 | 111 | 1.76 | 15.95 | 117 | 6.98 | 31.79 | 109 | 0.19 | 0.71 |
| Housing worker - face-to-face contacts in NHS setting | | 119 | 0.12 | 1.11 | 111 | 0.17 | 0.70 | 117 | 0.02 | 0.14 | 109 | 0.03 | 0.21 |
| Housing worker - face-to-face contacts in community | | 119 | 2.53 | 15.90 | 111 | 1.59 | 15.95 | 117 | 6.96 | 31.80 | 109 | 0.16 | 0.63 |
| Voluntary/Charity worker (e.g. advocacy) - phone contacts | | 118 | 0.03 | 0.28 | 111 | 0.04 | 0.23 | 117 | 0.03 | 0.16 | 108 | 0.03 | 0.29 |
| Voluntary/Charity worker (e.g. advocacy) - face-to-face contacts | | 118 | 0.30 | 1.34 | 111 | 0.43 | 2.32 | 117 | 0.24 | 1.21 | 109 | 2.19 | 17.87 |
| Voluntary/Charity worker (e.g. advocacy) - face-to-face contacts in NHS setting | | 118 | 0.08 | 0.42 | 110 | 0.31 | 2.05 | 117 | 0.04 | 0.30 | 109 | 0.10 | 0.74 |
| Voluntary/Charity worker (e.g. advocacy) - face-to-face contacts in community | | 118 | 0.20 | 1.26 | 110 | 0.12 | 1.15 | 117 | 0.19 | 1.17 | 109 | 2.09 | 17.87 |
| Alternative therapies (NHS) - NHS face-to-face contacts (not paid for) | | 121 | 0.32 | 2.30 | 112 | 0.18 | 1.89 | 118 | 0.24 | 2.40 | 109 | 0.01 | 0.10 |
| Alternative therapies (NHS) - face-to-face contacts in NHS setting | | 121 | 0.12 | 0.75 | 112 | 0.18 | 1.89 | 118 | 0.02 | 0.13 | 109 | 0.01 | 0.10 |
| Alternative therapies (NHS) - face-to-face contacts in community | | 121 | 0.20 | 2.18 | 112 | 0.00 | 0.00 | 118 | 0.22 | 2.39 | 109 | 0.00 | 0.00 |
| Alternative therapies (private)- private face-to-face contacts (paid for) | | 121 | 0.01 | 0.06 | 112 | 0.05 | 0.42 | 118 | 0.01 | 0.06 | 109 | 0.05 | 0.39 |
| Alternative therapies (private)- face-to-face contacts in private health care facility | | 121 | 0.00 | 0.00 | 112 | 0.05 | 0.42 | 118 | 0.00 | 0.00 | 109 | 0.04 | 0.38 |
| Alternative therapies (private) - face-to-face contacts in community | | 121 | 0.01 | 0.06 | 112 | 0.00 | 0.00 | 118 | 0.01 | 0.06 | 109 | 0.01 | 0.10 |
| Day centre (groups/programs, non-health care staff) - contact | | 120 | 2.76 | 17.82 | 111 | 2.58 | 8.83 | 117 | 1.82 | 7.76 | 108 | 2.36 | 12.13 |
| Day hospital (group therapies etc., health care staff, regular) - contact | | 120 | 0.70 | 3.87 | 111 | 1.73 | 12.61 | 117 | 2.32 | 8.96 | 108 | 0.88 | 3.92 |
| Drop-in centre (including street agencies) (informal) - contact | | 120 | 0.13 | 1.20 | 111 | 1.22 | 10.52 | 116 | 0.14 | 1.24 | 108 | 1.89 | 17.83 |
| Self-help group/support group - contact | | 120 | 0.23 | 2.19 | 111 | 0.41 | 2.91 | 117 | 0.26 | 2.28 | 108 | 0.27 | 2.52 |
| Attendance in other facility (social club, dinner club) - contact | | 120 | 0.71 | 6.61 | 111 | 0.67 | 6.83 | 117 | 0.29 | 2.26 | 108 | 0.06 | 0.58 |
| Psychiatric hospital inpatient - number of days in hospital | | 163 | 45.77 | 58.81 | 165 | 46.83 | 60.81 | 163 | 23.69 | 50.96 | 165 | 29.38 | 55.35 |
| Psychiatric hospital inpatient - number of days in PICU | | 163 | 3.89 | 22.95 | 165 | 2.65 | 17.50 | 163 | 6.02 | 29.28 | 165 | 3.37 | 21.08 |
| Day-patient hospital admission: accident and emergency - number of contacts | | 120 | 0.29 | 1.85 | 112 | 0.38 | 1.15 | 116 | 0.07 | 0.24 | 110 | 0.22 | 0.56 |

| **Supplementary Table 3: Mean health and social care costs and productivity and informal care costs per participant over the 12-month period, complete cases (£, year 2012/13 tariff)** | | | | | | | | | |
| --- | --- | --- | --- | --- | --- | --- | --- | --- | --- |
|  | **CTO** | | | **Non-CTO** | | | **CTO vs. Non-CTO** | | |
|  | **Mean** | **(SD)** | **N** | **Mean** | **(SD)** | **N** | **Mean difference** | **95% CI** | |
| **Complete case analysis** |  |  |  |  |  |  |  |  | |
| **Total medication costs** | 1,213.05 | (1,180.76) | 95 | 1,466.06 | (1,303.45) | 90 | -253.01 | -613.45 | 107.43 |
| Oral medication | 887.86 | (1,091.88) | 95 | 913.70 | (1,071.52) | 90 | -25.84 | -339.87 | 288.19 |
| Depot medication | 325.19 | (788.81) | 95 | 552.36 | (1,117.57) | 90 | -227.17 | -506.61 | 52.26 |
| **Total other health and social care costs** | 35,984.89 | (49,540.10) | 95 | 30,343.83 | (34,974.91) | 90 | 5,641.06 | -6,860.63 | 18,142.75 |
| Mental health community/outpatient | 2,186.01 | (2,109.39) | 95 | 1,907.73 | (2,224.22) | 90 | 278.28 | -350.35 | 906.90 |
| Mental health inpatient | 32,262.39 | (49,391.44) | 95 | 26,264.86 | (35,293.06) | 90 | 5,997.53 | -6,515.34 | 18,510.39 |
| Non-mental health outpatient | 103.81 | (363.68) | 95 | 140.83 | (300.17) | 90 | -37.03 | -134.05 | 60.00 |
| Non-mental health inpatient | 84.70 | (422.70) | 95 | 437.36 | (1,931.11) | 90 | -352.65 | -753.27 | 47.96 |
| Primary care | 151.89 | (270.11) | 95 | 227.92 | (467.73) | 90 | -76.02 | -186.11 | 34.06 |
| Social care | 1,196.09 | (1,944.48) | 95 | 1,365.14 | (2,401.07) | 90 | -169.04 | -801.30 | 463.22 |
| **Total health and social care costs** | 37,197.93 | (49,438.91) | 95 | 31,809.88 | (35,064.79) | 90 | 5,388.05 | -7,106.62 | 17,882.72 |
| **Indirect costs** | 4,384.41 | (14,425.84) | 59 | 1,619.28 | (3,982.76) | 57 | 2,765.13 | -1,093.82 | 6,624.07 |
| Lost productivity (sick leave) | 0 | - | 59 | 0 | - | 57 | 0 | - | - |
| Informal care | 4,384.41 | (14,425.84) | 59 | 1,619.28 | (3,982.76) | 57 | 2,765.13 | -1,093.82 | 6,624.07 |
| **TOTAL SOCIETAL COSTS** | 37,756.43 | (44,469.28) | 59 | 30,689.52 | (33,045.08) | 57 | 7,066.91 | -7,219.06 | 21,352.89 |

**Supplementary Figure 1: Cost-effectiveness acceptability curves: a) health and social care costs, imputed full dataset (n = 328); b) health and social care costs, complete case analysis (n = 121); c) societal costs, imputed full dataset (n = 328); d) societal costs, complete case analysis (n = 102)**

**a)**

**b)**

**
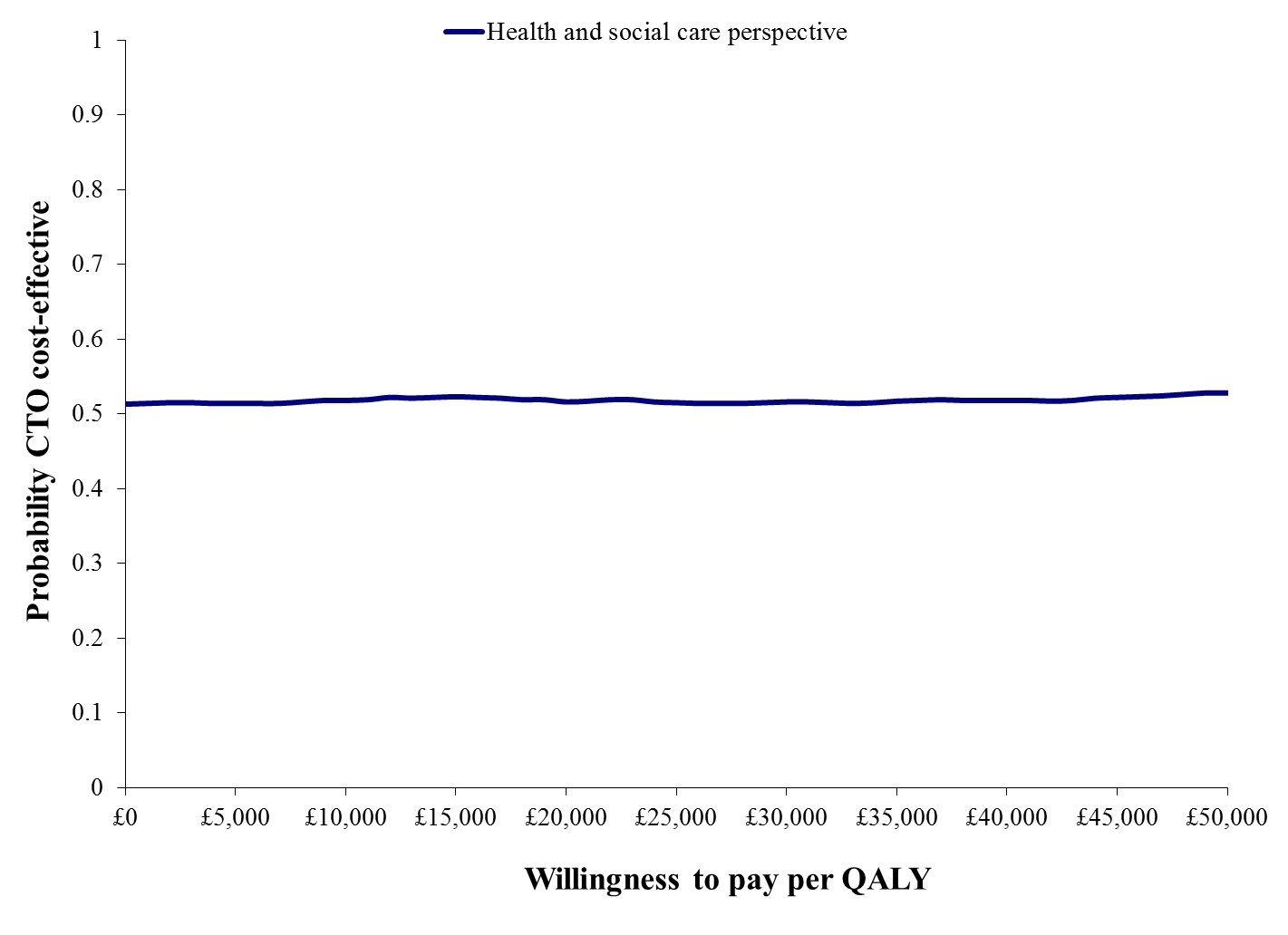

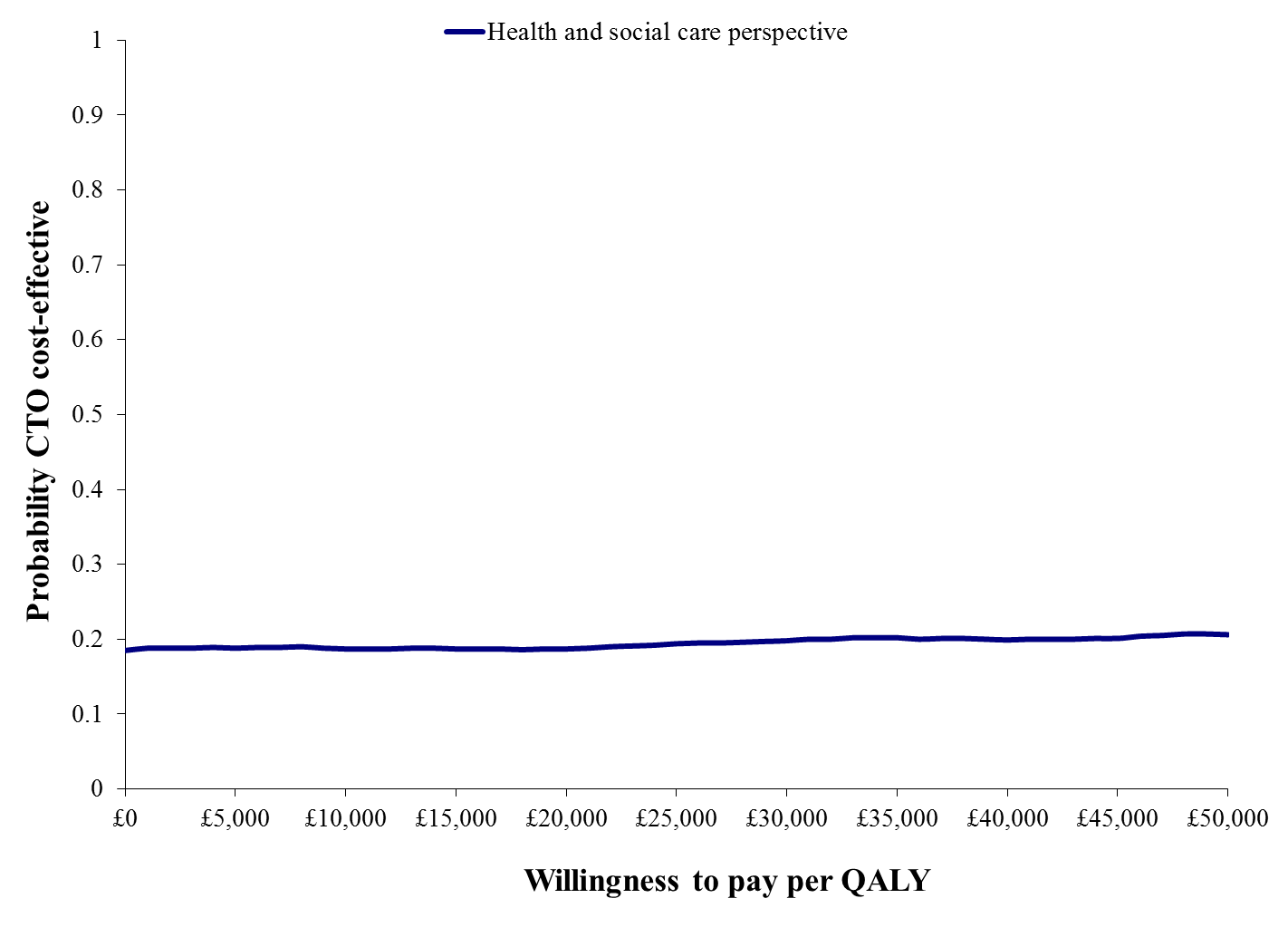
**

**d)**

**c)**


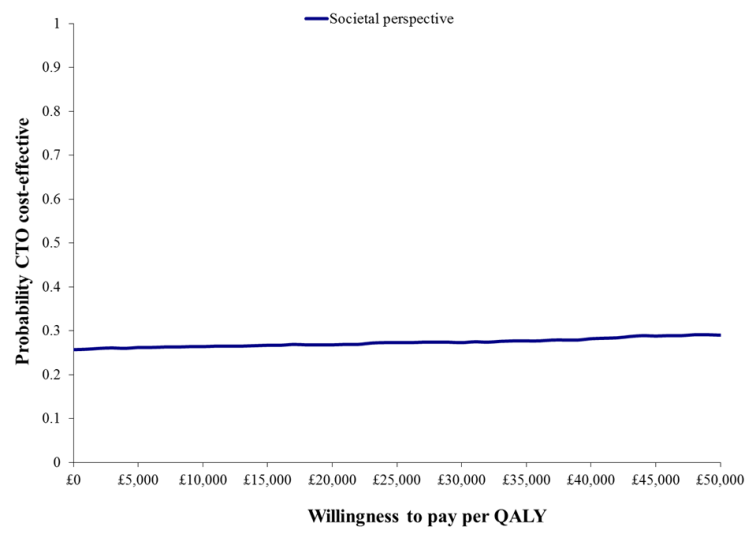
 **
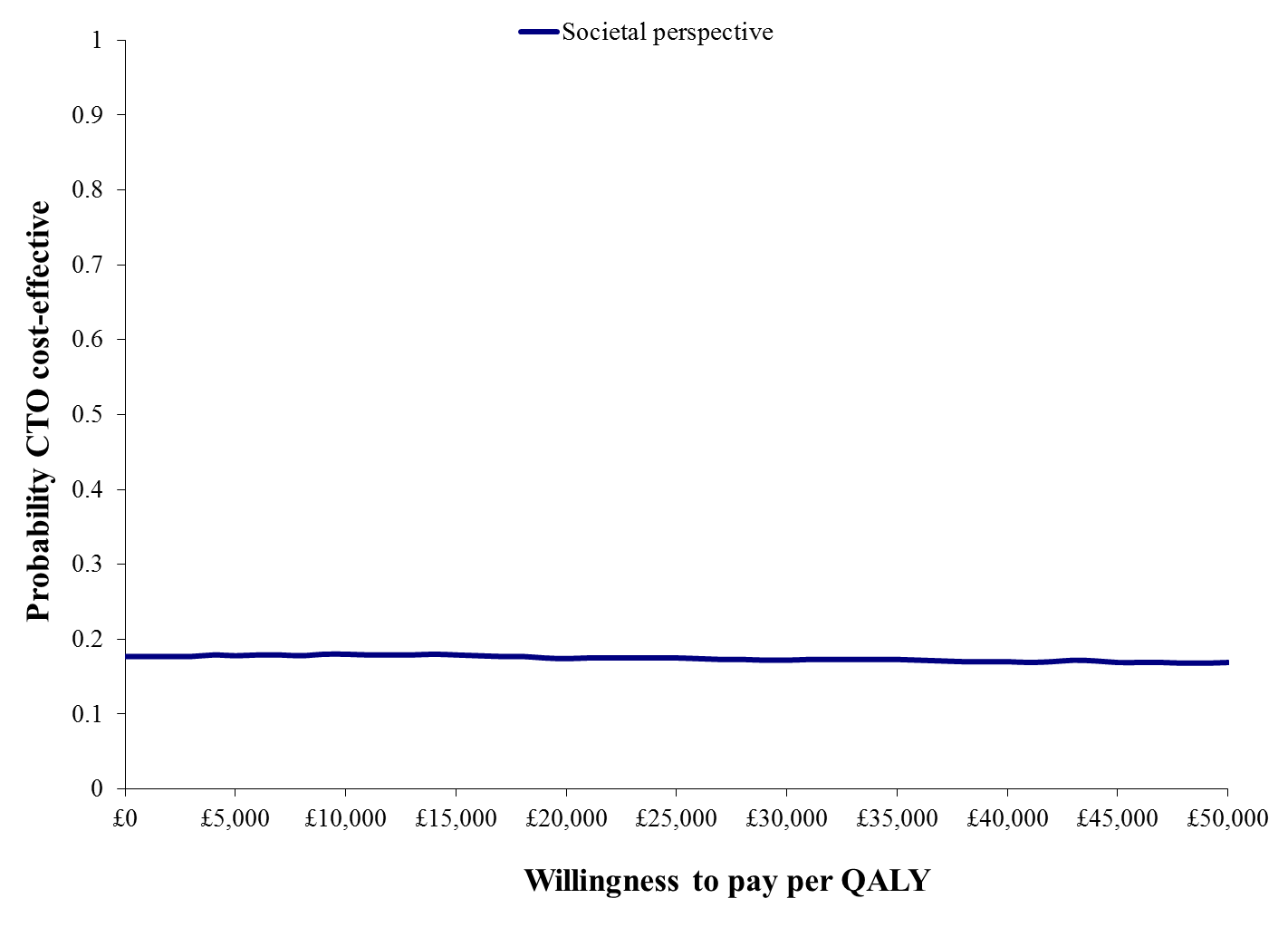
**
